# Structural Characterization and Antimicrobial Activities of 7*H*-Benzo[*h*]chromeno[2,3-*d*]pyrimidine and 14*H*-Benzo[*h*]chromeno[3,2-*e*][1,2,4]triazolo[1,5-*c*] pyrimidine Derivatives

**DOI:** 10.3390/molecules21111450

**Published:** 2016-11-01

**Authors:** Rawda M. Okasha, Fawzia F. Albalawi, Tarek H. Afifi, Ahmed M. Fouda, Al-Anood M. Al-Dies, Ahmed M. El-Agrody

**Affiliations:** 1 Chemistry Department, Faculty of Science, Taibah University, Al-Madinah Al-Munawarah 30002, Saudi Arabia; ffs_chem334@hotmail.com (F.F.A.); afifith@yahoo.com (T.H.A.); 2 Chemistry Department, Faculty of Science, King Khalid University, Abha 61413, P.O. Box 9004, Saudi Arabia; amfouda@hotmail.com (A.M.F.); nood-mansyaa@hotmail.com (A.M.A.-D.); 3 Chemistry Department, Faculty of Science, Al-Azhar University, Nasr City, Cairo 11884, Egypt; elagrody_am@yahoo.com

**Keywords:** 4*H*-benzochromene, 7*H*-benzochromenopyrimidine, 14*H*-benzochromenotriazolo-pyrimidine, antimicrobial activities, SAR

## Abstract

Three new series of chromene molecules have been synthesized in order to explore their antimicrobial activity. The series encompass 2-substituted 14-(4-halophenyl)-12-methoxy-14*H*-benzo[*h*]chromeno[3,2-*e*][1,2,4]-triazolo[1,5-*c*]pyrimidines **7a**–**o**, 9-benzylideneamino-7-(4-halo-phenyl)-5-methoxy-8-imino-7*H*-benzo-[*h*]chromeno[2,3-*d*]pyrimidines **8a**–**b** and 3-ethoxycarbonyl-14-(4-halophenyl)-12-methoxy-14*H*-benzo-[*h*]chromeno[3,2-*e*][1,2,4]triazolo[1,5-*c*]pyrimidine-2-one derivatives **12a**–**b**. The structure of these novel compounds were confirmed using IR, ^1^H- and ^13^C-NMR as well as MS spectroscopy. The new compounds were evaluated in vitro for their antimicrobial activity and it was demonstrated that 7*H*-benzochromenopyrimidine and derivatives of 14*H*-benzochromenotriazolopyrimidine exhibited the most promising antibacterial activities compared to the reference antimicrobial agents. The structure activity relationship (SAR) studies of the target compounds agreed with the in vitro essays and confirmed higher potent antimicrobial activity against some of the tested microorganisms.

## 1. Introduction

The development of antimicrobial agents is an area of great activity due to the emergence of multidrug resistance in common pathogens and the appearance of new infections. Infectious diseases are considered the second-leading cause of death worldwide; consequently, tremendous efforts have been made to develop new antimicrobial agents that are active especially against the drug resistant strains.

The majority of antimicrobial agents are diverse five- and six-membered heterocyclic molecules that play a crucial role in the metabolism of all living cells [1]. Moreover, a great deal of interest has been directed toward condensed ring systems due to their various types of physiological activities and the success in utilizing them as privileged medicinal scaffolds. In particular, fused chromene and benzochromene molecules have become some of the best potential candidates for pharmacological purposes due to their antimicrobial [2,3], antileishmanial [4,5], anticancer [6,7], antiproliferative [8], antioxidant [9,10], hypertensive [11], antitumor [12,13,14,15] effects and activities, as well as for the treatment of Alzheimer’s disease [16] and schizophrenia disorders [17].

Fused chromene ring systems also displayed blood platelet antiaggregating [18], antihistaminic [19], analgesic [20,21,22], hypolipidemic [23], DNA breaking and mutagenicity activities [24]. Furthermore, several reports have shown that 7*H*-benzo[*h*]chromeno[2,3-*d*]pyrimidine and 14*H*-benzo[*h*]chromeno[3,2-*e*][1,2,4]triazolo[1,5-*c*]pyrimidine moieties are promising and attractive scaffolds for the development of potent antimicrobial agents [4,25,26,27,28]. In addition, 7*H*-benzo[*h*]-chromeno[2,3-*d*]pyrimidine derivatives exhibit anticancer activities [29,30,31,32,33].

In continuation of the previous works [15,25,26,27,28,29,30,31,34,35,36,37], it seemed interesting to synthesize some new 7*H*-benzo[*h*]chromeno[2,3-*d*]-pyrimidine and 14*H*-benzo-14*H*-benzo[*h*]chromeno[3,2-*e*][1,2,4]-triazolo[1,5-*c*]pyrimidine derivatives and to appraise their antimicrobial activities. The structure-activity relationships (SAR) are discussed in this work to correlate between the substituent effects and the activities that aid in drug design.

## 2. Results and Discussion

### 2.1. Chemistry

**Scheme 1:**
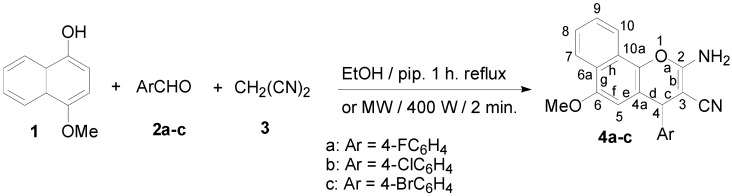
Synthesis of 2-amino-4-(4-halophenyl)-6-methoxy-4*H*-benzo[*h*]chromene-3-carbonitriles **4a**–**c**.

The first series of the new chromene compounds was synthesized via multi component reactions of 4-methoxy-1-naphthol (**1**) with 4-halobenzaldehydes **2a**–**c** and malononitrile (**3**) in ethanolic piperidine solution. The solution was refluxed for 1 h and gave the corresponding 2-amino-4-(4-halophenyl)-6-methoxy-4*H*-benzo[*h*]chromene-3-carbonitriles **4a**–**c**. The desired compounds were also obtained from the same precursors using microwave irradiation for 2 min. at 140 °C. These results are depicted in Scheme 1.

**Scheme 2:**
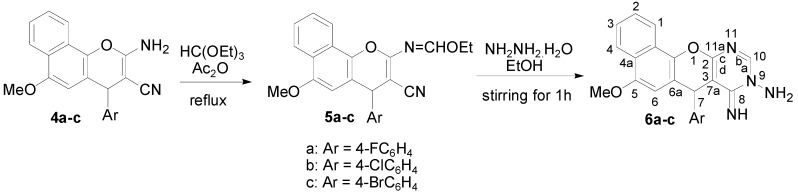
Synthesis of 2-ethoxymethyleneamino-4-(4-halophenyl)-6-methoxy-4*H*-benzo[*h*]chromene-3-carbonitriles (**5a**–**c**) and 9-amine-7-(4-halorophenyl)-5-methoxy-8-imino-7*H*-benzo[*h*]chromeno[2,3-*d*]pyrimidines (**6a**–**c**).

Treatment of compounds **4a**–**c** with triethyl orthoformate in acetic anhydride at reflux gave the 2-ethoxymethyleneamino-4-(4-halophenyl)-6-methoxy-4*H*-benzo[*h*]chromene-3-carbonitriles **5a**–**c**. Subsequent hydrazinolysis of the latter in ethanol under stirring at room temperature gave 9-amino-7-(4-halophenyl)-5-methoxy-8-imino-7*H*-benzo[*h*]chromeno[2,3-*d*] pyrimidines **6a**–**c**, as illustrated in Scheme 2.

**Scheme 3:**
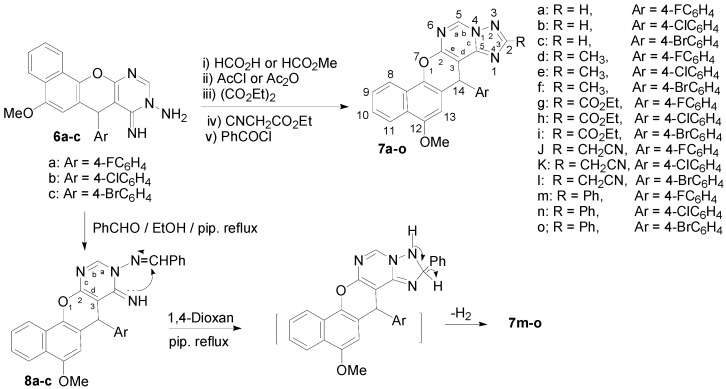
2-substituted 14-(4-halophenyl)-12-methoxy-14*H*-benzo[*h*]chromeno[3,2-*e*][1,2,4]triazolo[1,5-*c*]pyrimidines (**7a**–**o**) and 9-benzylideneamino-7-(4-halophenyl)-5-methoxy-8-imino-7*H*-benzo[*h*]chromeno[2,3-*d*]pyrimidines (**8a**–**c**).

The aminoimino compounds **6** proved to be useful intermediates for the synthesis of a variety of 2-substituted 14*H*-benzo[*h*]chromeno[3,2-*e*][1,2,4]triazolo[1,5-*c*]pyrimidine derivatives. Thus, treatment of the aminoimino compounds **6a**–**c** with either formic acid or methyl formate in benzene at reflux gave 14-(4-halophenyl)-12-methoxy-14*H*-benzo[*h*]chromeno[3,2-*e*]-[1,2,4]triazolo[1,5-*c*]-pyrimidines **7a**–**c**, whilst acylation of compounds **6a**–**c** with acetyl chloride or acetic anhydride gave the pentacyclic 2-methyltriazolopyrimidine derivatives **7d**–**f**. On the other hand, condensation of compounds **6a**–**c** with diethyl oxalate and ethyl cyanoacetate afforded 2-ethoxycarbonyltriazolo-pyrimidines **7g**–**i** and 2-cyanomethyltriazolopyrimidine derivatives **7j**–**l**, respectively, as shown in Scheme 3.

Aroylation of compounds **6a**–**c** with benzoyl chloride in refluxing dry benzene proceeded readily to give the 2-phenyltriazolopyrimidine derivatives **7m**–**o**, Scheme 3. Condensation of the aminoimino compounds **6a**–**c** with benzaldehyde in ethanolic piperidine solution under reflux gave the open chain product 9-benzylideneamino-7-(4-halophenyl)-5-methoxy-8-imino-7*H*-benzo[*h*]-chromeno[2,3-*d*]pyrimidines **8a**–**c**, Scheme 3. Compounds **7m**–**o** were also prepared by cyclization of compounds **8a**–**c** in 1,4-dioxane/piperdine solution under reflux [26] as confirmed by the m.p., mixed m.p., and their identical IR and MS spectra, Scheme 3.

**Scheme 4:**
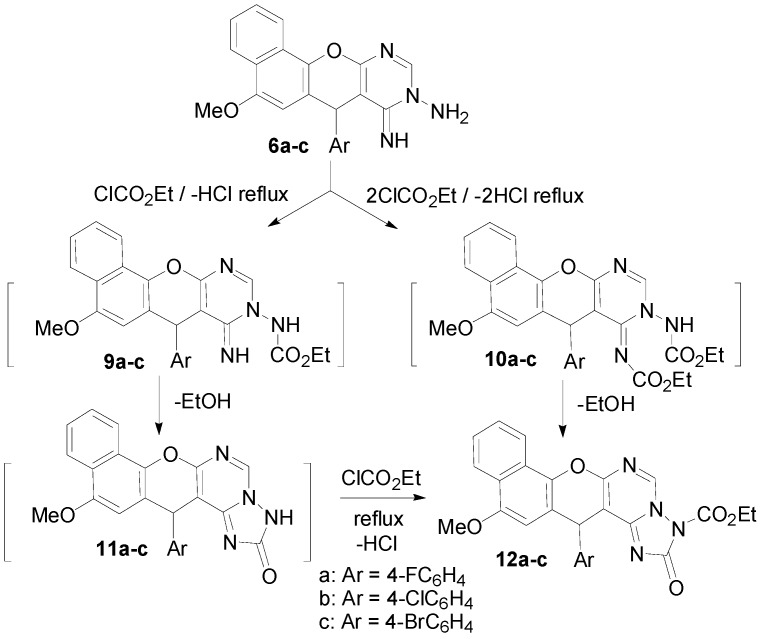
Synthesis of 3-ethoxycarbonyl-14-(4-halophenyl)-12-methoxy-14*H*-benzo[*h*]chromeno[3,2-*e*][1,2,4]triazolo[1,5-*c*]pyrimidine-2-ones (**12a**–**c**).

Interaction of the aminoimino compounds **6a**–**c** with ethyl chloroformate in dry benzene at reflux gave the corresponding 1:2 adducts, the 3-ethoxycarbonyl-14-(4-halophenyl)-12-methoxy-14*H*-benzo[*h*]-chromeno[3,2-*e*]-[1,2,4]triazolo[1,5-*c*]pyrimidine-2-ones **12a**–**c** instead of the 1:1 adducts, the triazolopyrimidin-2-one derivatives **11a**–**c**. These results are depicted in Scheme 4. The formation of **12a**–**c** is assumed to proceed via interaction of **6a**–**c** with one mole of ethyl chloroformate and elimination of HCl to yield the intermediates **9a**–**c**, which then cyclized to the non-isolable compounds **11a**–**c** via elimination of EtOH. The intermediates **11a**–**c** then reacted with another mole of ethyl chloroformate to eliminate HCl and give **12a**–**c**. Alternatively, interaction of **6a**–**c** with two moles of ethyl chloroformate could eliminate two HCl molecules and yield the intermediate bis-(ethoxycarbonyl) derivatives **10a**–**c**, which then cyclized to **12a**–**c** with elimination of an ethanol molecule (Scheme 4). The structures of **7**, **8** and **12** were established on the basis of IR, ^1^H-NMR, ^13^C-NMR and MS data. The 7-position of compounds **8** and the 14-position of compounds **7** and **12** are chiral centers, and all the reactions were monitored using the TLC technique.

### 2.2. Antibacterial Evaluation

The target compounds **7a**–**o**, **8a**–**c** and **12a**–**c** were tested in vitro for their antimicrobial activities by the agar diffusion method using Mueller-Hinton agar medium for bacteria and Sabouraud’s agar medium for fungi [32,33]. The tested microorganisms were obtained from the Regional Center for Mycology & Biotechnology (RCMP), Al-Azhar University.

**Figure 1:**
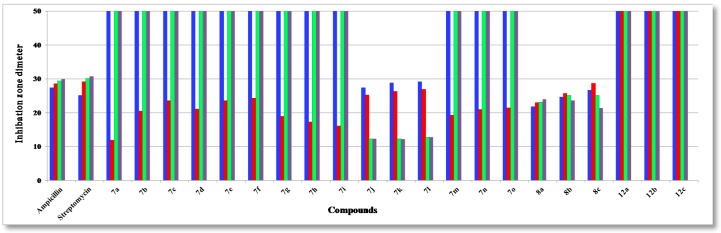
Antibacterial activity (Gram-positive) of the tested compounds compared to ampicillin and streptomycin: *S. aureus* (blue); *S. epidermidis* (red); *B.subtilis* (green) and *B. pumilus* (pale purple).

**Figure 2:**
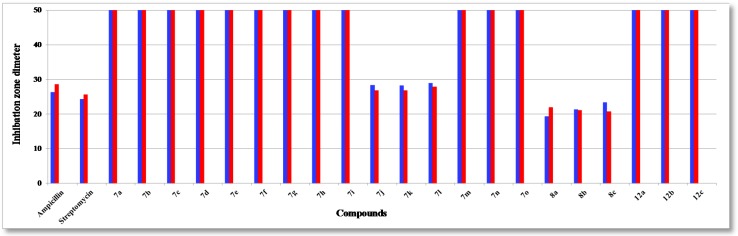
Antibacterial activity (Gram-negative) of tested compounds compared to ampicillin and streptomycin: *P. aeruginosa* (blue) and *E. coli* (red).

**Figure 3:**
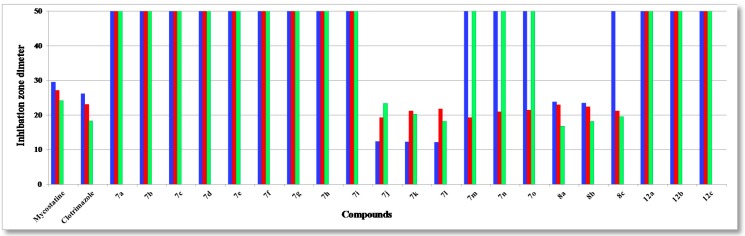
Antifungal activity of tested compounds compared to mycostatine and clotrimazole: *A. fumigatus* (blue); *C. albicans* (red) and *S. cerevisiae* (green).

The assayed collection included four Gram-positive species of pathogenic bacteria: *Staphylococcus aureus* (RCMB 000106), *Staphylococcus epidermidis* (RCMB 000107), *Bacillis subtilis* (RCMB 000108), *Bacillus pumilus* (RCMB 000109), and two Gram-negative ones: *Pseudomonas aeruginosa* (RCMB 000102), *Escherichia coli* (RCMB 000103), using two standard antibiotics, ampicillin, and streptomycin (25 µg/mL) as reference drugs, and three fungi: *Aspergillus fumigatus* (RCMB 002003), *Candida albicans* (RCMB 005002) and *Saccharomyces cerevisiae* (RCMB 006002), using two standard antibiotics, mycostatine and clotrimazole (25 µg/mL) as reference drugs. The mean zone of inhibition in mm ± standard deviation beyond the well diameter (6 mm) was determined using a 25 µg/mL concentration of the tested compounds. The inhibitory effects of the synthetic compounds against these organisms are given in Figure 1, Figure 2, and Figure 3 and Table 1.

### 2.3. SAR Studies

The structure activity relationship (SAR) studies of compounds **7a**–**o**, **8a**–**c** and **12a**–**c** revealed that compounds **8a**–**c** and **7j** with inhibitory effects of 21.8 ± 0.14, 24.6 ± 0.21, 26.6 ± 0.13 and 27.3 ± 0.47 µg/mL were 1.3, 1.1, 1.0, 1.0 times more active than the standard antibiotic ampicillin (27.4 ± 0.15 µg/mL)**, while compounds **8a**, **b** were 1.2 and 1.0 times more active than the standard antibiotic streptomycin (25.1 ± 0.18 µg/mL) against *S. aureus*, respectively, and other compounds showed almost equipotent activities or were inactive, implying that the 7*H*-benzo[*h*]chromeno-[2,3-*d*]-pyrimidine nucleus was more active than the 14*H*-benzo[*h*]chromeno[3,2-*e*][1,2,4]triazolo-[1,5-*c*]-pyrimidine one and grafting a lipophilic hydrophobic group (=NH-8, –N=CHPh-9) or halogen (F > Cl > Br) on the 4-position of the phenyl group at the 7-position of the 7*H*-benzo[*h*]chromeno-[2,3-*d*]pyrimidine moiety is more beneficial than a CH_2_CN group at the 2-position of a 14*H*-benzo[*h*]chromeno[3,2-*e*][1,2,4]-triazolo[1,5-*c*]pyrimidine. Compounds **7a**,**i**,**h**,**g**,**m**,**b**,**n**,**d**,**o**, **8a**, **7c**,**e**,**f**,**j**, **8b**, **7k**,**l**, **8c** were found to be the most potent against *S. epidermidis*, with inhibitory effects ranging from 11.9 to 28.5 µg/mL compared to standard antibiotics ampicillin and streptomycin (28.6 ± 0.5 and 29.1 ± 0.3 µg/mL), suggesting that the 14*H*-benzo[*h*]chromeno[3,2-*e*][1,2,4]triazolo[1,5-*c*]pyrimidine nucleus was more effective than the 7*H*-benzo[*h*]chromeno[2,3-*d*]pyrimidine nucleus. Moreover, the non-substituted triazolo ring at the 2-postion is better than a hydrophobic moiety like a CO_2_Et, Ph, Me and CH_2_CN group. In addition, compounds **7k**,**j**,**l** and **8a**–**c**) showed good activity against *B. subtilis* with inhibitory effects ranging 12.2–25.1 µg/mL as compared to the standard antibiotics ampicillin and streptomycin (29.4 ± 0.7 and 30.1 ± 0.9 µg/mL), while compounds **7k**,**j**,**l** and **8c**,**b**,**a** with inhibitory effects ranging 12.2–24.0 µg/mL, exhibited good activity against *B. pumilus* as compared to the standard antibiotics ampicillin and streptomycin (29.9 ± 0.3 and 30.8 ± 0.4 µg/mL). These results implied that 14*H*-benzo[*h*]chromeno[3,2-*e*][1,2,4]triazolo[1,5-*c*]pyrimidine nucleus with hydrophobic group CH_2_CN group at 2-position was more beneficial than 7*H*-benzo[*h*]chromeno[2,3-*d*]pyrimidine nucleus with a lipophilic hydrophobic groups (=NH-8, –N=CHPh-9). Furthermore, compounds **8a**–**c** exhibited good activities against *P. aeruginosa*, with inhibitory effects ranging from 19.3–23.3 µg/mL, as compared to the standard antibiotics ampicillin and streptomycin (26.3 ± 0.3 and 24.3 ± 0.8 µg/mL), while compounds **8c**,**b**,**a** and **7k**,**j**,**l** showed high activities against *E. coli*, with inhibitory effects ranging from 20.7–27.8 µg/mL as compared to the standard antibiotics ampicillin and streptomycin (28.5 ± 0.1 and 25.6 ± 0.4 µg/mL), suggesting that a 7*H*-benzo[*h*]chromeno[2,3-*d*]pyrimidine nucleus with a lipophilic hydrophobic group (=NH-8, –N=CHPh-9) was more effective than a 14*H*-benzo[*h*]chromeno[3,2-*e*][1,2,4]-triazolo[1,5-*c*]pyrimidine nucleus with a hydrophobic CH_2_CN group at the 2-position. Finally, compounds **7l**,**k**,**j**, **7j**–**l** and **8c**,**b**,**a**)with inhibitory effects ranging from 12.1–12.3, 19.3–21.2, 22.1–23.8 and 21.7–22.9 µg/mL, respectively, showed high activity against *A. fumigatus* and *C. albicans* as compared to the standard antibiotics mycostatine (29.5 ± 0.1 and 27.1 ± 0.1 µg/mL) and clotrimazole (26.1 ± 0.1 and 23.1 ± 0.3 µg/mL), respectively, implying that the 14*H*-benzo[*h*]chromeno[3,2-*e*][1,2,4]-triazolo[1,5-*c*]pyrimidine nucleus with a hydrophobic CH_2_CN group at the 2-position was more active than a 7*H*-benzo[*h*]chromeno[2,3-*d*]pyrimidine nucleus with a lipophilic hydrophobic group (=NH-8, –N=CHPh-9), while compounds **8a**–**c** and **7l**,**k**,**j** with inhibitory effects between 16.8–19.5 and 18.2–23.3 µg/mL, respectively, are more effective against *S. cerevisiae* as compared to the standard antibiotic mycostatine (24.1 ± 0.3 µg/mL) and compounds **8a**,**b** and **7l** (16.8–18.2 µg/mL) are more active than the standard antibiotic clotrimazole (18.3 ± 0.1 µg/mL). The rest of the compounds showed almost equipotent and moderate activities or were inactive.

## 3. Experimental Section

### 3.1. General Information

Commercial-grade solvents and reagents were purchased from Sigma-Aldrich (St. Louis, MO, USA) and used without further purification. Melting points were measured with a Stuart Scientific (Stone, Staffordshire, UK) apparatus and are uncorrected. IR spectra were determined as KBr pellets on a Jasco FT/IR 460 plus spectrophotometer (Jasco, Tokoyo, Japan). ^1^H-NMR (500 MHz) and ^13^C-NMR (125 MHz) spectra were recorded using a Bruker AV 500 MHz spectrometer (Bruker, Billerica, MA, USA). Chemical shifts (δ) are expressed in parts per million (ppm). The ^1^H-NMR and ^13^C-NMR spectra of the compounds are provided in the Supplementary Material. The MS were measured using a Shimadzu GC/MS-QP5050A spectrometer (Shimadzu, Tokoyo, Japan). Elemental analyses were carried out at the Regional Centre for Mycology & Biotechnology (RCMP, Al-Azhar University, Cairo, Egypt) and the results were within ±0.3% of calculated values. Analytical thin layer chromatography (TLC) on silica gel precoated F_254_ plates (Merck, Billerica, MA, USA) was used to check the purity of the final compounds and intermediates.

### 3.2. Synthesis

#### 3.2.1. Starting Materials

2-Amino-4-(4-fluoro/chloro/bromophenyl)-6-methoxy-4*H*-benzo[h]chromene-3-carbonitriles **4a**–**c**, 2-ethoxymethyleneamino-4-(4-fluoro/chloro/bromophenyl)-6-methoxy-4*H*-benzo[*h*]chromene-3-carbonitriles **5a**–**c** and 9-amino-7-(4-fluoro/chloro/bromophenyl)-5-methoxy-8-imino-7*H*-benzo[*h*]-chromeno[2,3-*d*]pyrimidines **6a**–**c** were prepared by reported methods ([34,35,36,37], [29,30,31] and [29,30,31], respectively).

#### 3.2.2. General Procedure for the Synthesis of Compounds **7a**–**c**

A mixture of aminoimino compound **6a**–**c** (3.88, 4.04, or 4.49 g, respectively, 0.01 mol) and formic acid or methyl formate (0.46 g or 0.6 g, 0.01 mol) in dry benzene (30 mL) was refluxed for 5 h. The solvent was extracted and the resulting products were recrystallized from ethanol/1,4-dioxane to give **7a**–**c**. The physical data of the compounds **7a**–**c** are as follows:

*14-(4-Fluorophenyl)-12-methoxy-14H-benzo[h]chromeno[3,2-e][1,2,4]triazolo[1,5-c]pyrimidine* (**7a**). Pale yellow crystals. Yield: 79%; m.p. 262–263 °C; IR (KBr, cm*^−^*^1^): ν 3053, 3010 (CH-arom.), 2959, 2848 (CH-aliph.), 1652 (C=N); ^1^H-NMR (DMSO-*d*_6_) δ 3.87 (s, 3H, OCH_3_), 5.34 (s, 1H, H-14), 6.68–8.22 (m, 11H, Ar-H, H-2, H-5); ^13^C-NMR (DMSO-*d*_6_) δ 39.02 (C-14), 55.75 (CH_3_), 98.75 (C-14a), 103.59 (C-13), 115.22 (Ar-C), 120.66 (C-13a), 121.65 (C-8), 124.02 (C-11), 124.39 (C-10), 126.18 (C-11a), 127.37 (C-9), 129.71 (C-8a), 129.77 (Ar-C), 136.67 (C-5), 140.25 (Ar-C), 141.36, (C-8b), 146.79 (C-14b), 151.42 (C-12), 155.91 (C-2), 160.01 (Ar-C), 161.94 (C-6a); MS (*m/z*), 398 (M^+^, 11.46) with a base peak at 278 (100); C_23_H_15_FN_4_O_2_ (398.39): calcd; % C: 69.34, % H: 3.80, % N: 14.06; found; % C: 69.40, % H: 3.84, % N: 14.10.

*14-(4-Chlorophenyl)-12-methoxy-14H-benzo[h]chromeno[3,2-e][1,2,4]triazolo[1,5-c]pyrimidine* (**7b**). Pale yellow crystals. Yield: 92%; m.p. 242–243 °C; IR (KBr, cm*^−^*^1^): ν 3071, 3011 (CH-arom.), 2953, 2912, 2862, 2828 (CH-aliph.), 1651 (C=N); ^1^H-NMR (DMSO-*d*_6_) δ 3.86 (s, 3H, OCH_3_), 5.34 (s, 1H, H-14), 6.68–8.22 (m, 10H, Ar-H, H-5), 8.34 (s, 1H, H-2); ^13^C-NMR (DMSO-*d*_6_) δ 39.91 (C-14), 55.73 (CH_3_), 98.73 (C-14a), 103.51 (C-13), 120.66 (C-13a), 121.64 (C-8), 124.00 (C-11), 124.41 (C-10), 126.20 (C-11a), 127.37 (C-9), 128.28 (C-8a), 128.38 (Ar-C), 129.77 (Ar-C), 133.20 (Ar-C), 137.37 (C-5), 141.64 (Ar-C), 143.37 (C-8b), 148.77 (C-14b), 151.42 (C-12), 155.92 (C-2), 166.92 (C-6a); MS (*m/z*), 416 (M^+^ + 2, 19.25), 414 (M^+^, 4.67) with a base peak at 62 (100); C_23_H_15_ClN_4_O_2_ (414.84): calcd; % C: 66.59, % H: 3.64, % N: 13.51; found; % C: 66.64, % H: 3.70, % N: 13.56.

*14-(4-Bromophenyl)-12-methoxy-14H-benzo[h]chromeno[3,2-e][1,2,4]triazolo[1,5-c]pyrimidine* (**7c**). Pale yellow crystals. Yield: 83%; m.p. 232–233 °C; IR (KBr, cm*^−^*^1^): ν 3071, 3007 (CH-arom.), 2962, 2915, 2895 (CH-aliph.), 1652 (C=N); ^1^H-NMR (DMSO-*d*_6_) δ 3.87 (s, 3H, OCH_3_), 5.32 (s, 1H, H-14), 6.67–8.22 (m, 10H, Ar-H, H-5), 8.95 (s, 1H, H-2); ^13^C-NMR (DMSO-*d*_6_) δ 40.03 (C-14), 55.75 (CH_3_), 96.96 (C-14a), 103.63 (C-13), 120.03 (Ar-C), 120.67 (C-13a), 121.66 (C-8), 124.01 (C-11), 124.44 (C-10), 126.21 (C-11a), 127.38 (C-9), 130.16 (C-8a), 131.39 (Ar-C), 133.53 (Ar-C), 137.56 (C-5), 141.70, (C-8b), 143.24 (Ar-C), 151.44 (C-14b), 152.44 (C-12), 155.94 (C-2), 161.44 (C-6a), 163.94 (C-6a); MS (*m/z*), 460 (M^+^ + 2, 6.03), 458 (M^+^, 7.68) with a base peak at 279 (100); C_23_H_15_BrN_4_O_2_ (459.29): calcd; % C: 60.15, % H: 3.29, % N: 12.20; found; % C: 60.20, % H: 3.34, % N: 12.27.

#### 3.2.3. General Procedure for the Preparation of Compounds **7d**–**f**

A mixture of aminoimino compounds **6a**–**c** (3.88, 4.04, or 4.49 g, respectively, 0.01 mol) and acetyl chloride or acetic anhydride (30 mL) was refluxed for 2 h. The solvent was extracted and the resulting product was recrystallized from 1,4-dioxan to give **7d**–**f**. The physical data of the compounds **7d**–**f** are as follows:

*14-(4-Fluorophenyl)-2-methyl-12-methoxy-14H-benzo[h]chromeno[3,2-e][1,2,4]triazolo[1,5-c]pyrimidine* (**7d**). Colourless crystals. Yield: 81%; m.p. 252–253 °C; IR (KBr, cm*^−^*^1^): ν 3068, 3035, 3006 (CH-arom.), 2967, 2938, 2848 (CH-aliph.), 1623 (C=N); MS (*m/z*), 412 (M^+^, 77.98) with a base peak at 318 (100); C_24_H_17_FN_4_O_2_ (412.42): calcd; % C: 69.89, % H: 4.15, % N: 13.59; found; % C: 69.94, % H: 4.17, % N: 13.61.

*14-(4-Chlorophenyl)-2-methyl-12-methoxy-14H-benzo[h]chromeno[3,2-e][1,2,4]triazolo[1,5-c]pyrimidine* (**7e**). Pale yellow crystals. Yield: 89%; m.p. 238–239 °C; IR (KBr, cm*^−^*^1^): ν 3071, 3025 (CH-arom.), 2970, 2937, 2845 (CH-aliph.), 1632 (C=N); ^1^H-NMR (DMSO-*d*_6_) δ 2.46 (s, 3H, CH_3_), 3.91 (s, 3H, OCH_3_), 5.71 (s, 1H, H-14), 6.68–8.35 (m, 9H, Ar-H), 9.59 (s, 1H, H-5); ^13^C-NMR (DMSO-*d*_6_) δ 14.28 (CH_3_), 40.05 (C-14), 55.88 (CH_3_), 103.76 (C-13), 117.75 (C-13a), 119.77 (C-14a), 121.75 (C-8), 124.04 (C-11), 124.59 (C-10), 126.38 (C-11a), 127.57 (C-9), 128.28 (C-8a), 129.51 (Ar-C), 130.64 (Ar-C), 135.92 (Ar-C), 136.52 (C-5), 139.47 (C-8b), 146.01 (Ar-C), 148.54 (C-14b), 152.76 (C-12), 153.91 (C-2), 165.18 (C-6a), 151.44 (C-14b), 152.38 (C-12), 155.94 (C-2), 161.94 (C-6a), 163.90 (C-6a); MS (*m/z*), 430 (M^+^ + 2, 19.45), 428 (M^+^, 57.92) with a base peak at 51 (100); C_24_H_17_ClN_4_O_2_ (428.87): calcd; % C: 67.21, % H: 4.00, % N: 13.06; found; % C: 67.30, % H: 4.09, % N: 13.10.

*14-(4-Bromophenyl)-2-methyl-12-methoxy-14H-benzo[h]chromeno[3,2-e][1,2,4]triazolo[1,5-c]pyrimidine* (**7f**). Colourless crystals. Yield: 84%; m.p. 243–244 °C; IR (KBr, cm*^−^*^1^): ν 3060, 3021, 3006 (CH-arom.), 2958, 2932, 2848 (CH-aliph.), 1637 (C=N); ^1^H-NMR (DMSO-*d*_6_) δ 2.41 (s, 3H, CH_3_), 3.97 (s, 3H, OCH_3_), 5.33 (s, 1H, H-14), 6.17–8.45 (m, 9H, aromatic), 9.76 (s, 1H, H-5); ^13^C-NMR (DMSO-*d*_6_) δ 14.53 (CH_3_), 38.58 (C-14), 55.99 (CH_3_), 96.80 (C-14a), 103.39 (C-13), 120.08 (Ar-C), 120.57 (C-13a), 121.73 (C-8), 124.05 (C-11), 124.56 (C-10), 128.91 (C-11a), 129.62 (C-9), 130.24 (C-8a), 131.14 (Ar-C), 134.25 (Ar-C), 137.62 (C-5), 143.90 (C-8b), 144.55 (Ar-C), 151.70 (C-14b), 156.57 (C-12), 158.48 (C-2), 163.90 (C-6a); MS (*m/z*), 474 (M^+^ + 2, 12.19), 472 (M^+^, 13.44) with a base peak at 75 (100); C_24_H_17_BrN_4_O_2_ (473.32): calcd; % C: 60.90, % H: 3.62, % N: 11.84; found; % C: 60.96, % H: 3.66, % N: 11.88.

#### 3.2.4. General Procedure for the Preparation of Compounds **7g**–**i**

A mixture of aminoimino compounds **6a**–**c** (3.88, 4.04, or 4.49 g, respectively, 0.01 mol) and diethyl oxalate (1.46 g, 0.01 mol) in ethanol (30 mL) was refluxed for 2 h. The solvent was extracted and the resulting product was recrystallized from ethanol/1,4-dioxane to give **7g**–**i**. The physical data of the compounds **7g**–**i** are as follows:

*2-Ethoxycarbonyl-14-(4-fluorophenyl)-12-methoxy-14H-benzo[h]chromeno[3,2-e][1,2,4]-triazolo[1,5-c]-pyrimidine* (**7g**). Yellow crystals. Yield: 75%; m.p. 299–300 °C; IR (KBr, cm*^−^*^1^): ν 3067 (CH-arom.), 2995, 2987, 2939 (CH-aliph.), 1639 (C=N), 1743 (CO); ^1^H-NMR (DMSO-*d*_6_) δ 1.36 (t, 3H, CH_3_, *J* = 7.2 Hz), 3.93 (s, 3H, OCH_3_), 4.42 (q, 2H, CH_2,_ *J =* 7.2 Hz), 5.97 (s, 1H, H-14), 6.63–8.64 (m, 9H, Ar-H), 9.82 (s, 1H, H-5); ^13^C-NMR (DMSO-*d*_6_) δ 14.00 (CH_3_), 37.95 (C-14), 55.98 (CH_3_), 61.92 (CH_2_), 102.68 (C-13), 103.55 (C-13a), 115.73 (Ar-C), 117.90 (C-14a), 121.79 (C-8), 123.89 (C-11), 124.62 (C-10), 126.68 (C-11a), 127.95 (C-9), 128.30 (C-8a), 129.95 (Ar-C), 138.62 (C-5), 139.95 (Ar-C), 141.32 (C-8b), 153.06 (C-14b), 154.57 (C-12), 157.43 (C-2), 159.46 (C-6a), 160.53 (Ar-C), 164.55 (CO); MS (*m/z*), 470 (M^+^, 17.39) with a base peak at 260 (100); C_26_H_19_FN_4_O_4_ (470.45): calcd; % C: 66.38, % H: 4.07, % N: 11.91; found; % C: 66.42, % H: 4.11, % N: 12.00.

*2-Ethoxycarbonyl-14-(4-chlorophenyl)-12-methoxy-14H-benzo[h]chromeno[3,2-e][1,2,4]-triazolo[1,5-c]-pyrimidine* (**7h**). Colourless powder. Yield: 87%; m.p. 248–249 °C; IR (KBr, cm*^−^*^1^): ν 3071 (CH-arom.), 2979, 2945, 2920 (CH-aliph.), 1625 (C=N), 1747 (CO); MS (*m/z*), 488 (M^+^ + 2, 1.15), 486 (M^+^, 3.52) with a base peak at 111 (100); C_26_H_19_ClN_4_O_4_ (486.91): calcd; % C: 64.14, % H: 3.93, % N: 11.51; found; % C: 64.09, % H: 3.89, % N: 11.47.

*2-Ethoxycarbonyl-14-(4-bromophenyl)-12-methoxy-14H-benzo[h]chromeno[3,2-e][1,2,4]-triazolo[1,5-c]pyri-midine* (**7i**). Colourless needles. Yield: 80%; m.p. 288–289 °C; IR (KBr, cm*^−^*^1^): ν 3067, 3025, 3001 (CH-arom.), 2948, 2845 (CH-aliph.), 1626 (C=N), 1747 (CO); ^1^H-NMR (DMSO-*d*_6_) δ 1.52 (t, 3H, CH_3_, *J =* 7.2 Hz), 3.95 (s, 3H, OCH_3_), 4.59 (q, 2H, CH_2,_ *J =* 7.2 Hz), 5.93 (s, 1H, H-14), 6.43–8.51 (m, 9H, aromatic), 9.24 (s, 1H, H-5); ^13^C-NMR (DMSO-*d*_6_) δ 14.46 (CH_3_), 40.84 (C-14), 56.05 (CH_3_), 63.02 (CH_2_), 102.70 (C-13), 103.49 (C-13a), 116.04 (C-14a), 121.80 (C-8), 121.47 (Ar-C), 122.47 (C-11), 125.00 (C-10), 125.99 (C-11a), 126.86 (C-9), 127.90 (C-8a), 130.50 (Ar-C), 132.12 (Ar-C), 139.20 (C-5), 139.43 (C-8b), 142.16 (Ar-C), 153.54 (C-14b), 155.06 (C-2), 153.81 (C-12), 158.78 (C-6a), 159.93 (CO); MS (*m/z*), 532 (M^+^ + 2, 1.19), 530 (M^+^, 1.24) with a base peak at 76 (100); C_26_H_19_BrN_4_O_4_ (531.36): calcd; % C: 58.77, % H: 3.60, % N: 10.54; found; % C: 58.81, % H: 3.66, % N: 10.60.

#### 3.2.5. General Procedure for the Preparation of Compounds **7j**–**l**

A mixture of aminoimino compounds **6a**–**c** (3.88, 4.04, or 4.49 g, respectively, 0.01 mol) and ethyl cyanoacetate (1.13 g, 0.01 mol) in ethanol (30 mL) was refluxed for 2 h. (TLC monitoring). The solvent was extracted and the resulting product was recrystallized from ethanol/1,4-dioxane to give **7j**–**l**. The physical data of the compounds **7j**–**l** are as follows:

*2-Cyanomethyl-14-(4-fluorophenyl)-12-methoxy-14H-benzo[h]chromeno[3,2-e][1,2,4]-triazolo[1,5-c]-pyrimidine* (**7j**). Pale yellow crystals. Yield: 90%; m.p. >300 °C; IR (KBr, cm*^−^*^1^): ν 3065, 3026 (CH-arom.), 2983, 2940 (CH-aliph.), 2260 (CN), 1631 (C=N); ^1^H-NMR (DMSO-*d*_6_) δ 3.45 (s, 2H, CH_2_), 3.93 (s, 3H, OCH_3_), 5.62 (s, 1H, H-14), 6.63–8.32 (m, 9H, aromatic), 8.55 (s, 1H, H-5); ^13^C-NMR (DMSO-*d*_6_) δ 25.07 (CH_2_), 38.06 (C-14), 55.97 (CH_3_), 97.29 (C-13), 102.71 (C-13a), 116.50 (Ar-C), 117.41 (CN), 117.84 (C-14a), 120.64 (C-8), 121.79 (C-11), 123.90 (C-10), 124.63 (C-11a), 126.67 (C-9), 127.92 (C-8a), 130.00 (Ar-C), 138.65 (C-5), 140.36 (Ar-C), 151.34 (C-8b), 152.32 (C-14b), 153.37 (C-2), 159.14 (C-12), 160.80 (Ar-C), 167.38 (C-6a); MS (*m/z*), 437 (M^+^, 54.05) with a base peak at 252 (100); C_25_H_16_FN_5_O_2_ (437.43): calcd; % C: 68.64, % H: 3.69, % N: 16.01; found; % C: 68.70, % H: 3.74, % N: 16.08.

*2-Cyanomethyl-14-(4-chlorophenyl)-12-methoxy-14H-benzo[h]chromeno[3,2-e][1,2,4]-triazolo[1,5-c]-pyrimidine* (**7k**). Pale yellow powder. Yield: 83%; m.p. 240–241 °C; IR (KBr, cm*^−^*^1^): ν 3062, 3012 (CH-arom.), 2962, 2905, 2890 (CH-aliph.), 2258 (CN), 1624 (C=N); MS (*m/z*), 455 (M^+^ + 2, 8.15), 453 (M^+^, 25.25) with a base peak at 343 (100); C_25_H_16_ClN_5_O_2_ (453.88): calcd; % C: 66.16, % H: 3.55, % N: 15.43; found; % C: 66.55, % H: 3.49, % N: 15.38.

*2-Cyanomethyl-14-(4-bromophenyl)-12-methoxy-14H-benzo[h]chromeno[3,2-e][1,2,4]-triazolo[1,5-c]-pyrimidine* (**7l**). Colourless needles. Yield: 75%; m.p. 232–233 °C; IR (KBr, cm*^−^*^1^): ν 3077 (CH-arom.), 2959, 2925 (CH-aliph.), 2255 (CN), 1626 (C=N); ^1^H-NMR (DMSO-*d*_6_) δ 3.45 (s, 2H, CH_2_), 3.93 (s, 3H, OCH_3_), 5.61 (s, 1H, H-14), 6.63–8.26 (m, 9H, aromatic), 8.56 (s, 1H, H-5); ^13^C-NMR (DMSO-*d*_6_) δ 25.06 (CH_2_), 40.04 (C-14), 55.98 (CH_3_), 96.93 (C-13), 102.64 (C-13a), 117.45 (CN), 117.83 (C-14a), 120.53 (Ar-C), 120.79 (C-8), 121.79 (C-11), 123.88 (C-10), 124.62 (C-11a), 126.72 (C-9), 127.95 (C-8a), 129.88 (Ar-C), 131.76 (Ar-C), 136.96 (C-5), 141.71 (Ar-C), 151.41 (C-8b), 152.35 (C-14b), 153.38 (C-2), 159.18 (C-12), 167.38 (C-6a); MS (*m/z*), 499 (M^+^ + 2, 32.02), 497 (M^+^, 33.62) with a base peak at 342; C_25_H_16_BrN_5_O_2_ (498.33): calcd; % C: 60.25, % H: 3.24, % N: 14.05; found; % C: 60.31, % H: 3.37, % N: 14.10.

#### 3.2.6. General Procedure for the Preparation of Compounds **7m**–**o**

*Method A*: A mixture of aminoimino compound **6a**–**c** (3.88, 4.04, or 4.49 g, respectively, 0.01 mol) and benzoyl chloride (0.01 mol) in dry benzene (30 mL) was refluxed for 5 h. The solvent was removed under reduced pressure and the resulting solids were recrystallized from ethanol/1,4-dioxane to give **7m**–**o**.

*Method B*: A mixture of compounds **8a**–**c** (4.76, 4.92 or 5.37 g, respectively, 0.01 mol), dioxane (20 mL) and piperidine (0.5 mL) was refluxed for 2 h. The solvent was extracted and the resulting product was recrystallized from ethanol/1,4-dioxane to give **7m**–**o** in 60% yield (as verified by m.p., mixed m.p., identical IR and MS spectrum).

The physical data of the compounds **7m**–**o** are as follows:

*14-(4-Fluorophenyl)-2-phenyl-12-methoxy-14H-benzo[h]chromeno[3,2-e][1,2,4]triazolo[1,5-c]pyrimidine* (**7m**). Colourless needles. Yield: 67%; m.p. 248–249 °C; IR (KBr, cm*^−^*^1^): ν 3058, 3001 (CH-arom.), 2967, 2941 (CH-aliph.), 1625 (C=N); ^1^H-NMR (DMSO-*d*_6_) δ 3.90 (s, 3H, OCH_3_), 5.96 (s, 1H, H-14), 6.85–8.35 (m, 14H, aromatic), 9.69 (s, 1H, H-5); ^13^C-NMR (DMSO-*d*_6_) δ 40.03 (C-14), 55.88 (CH_3_), 101.24 (C-13), 103.72 (C-13a), 115.22 (Ar-C), 117.30 (C-14a), 120.66 (C-8), 121.75 (C-11), 124.07 (C-10), 124.69 (C-11a), 126.50 (C-9), 127.64 (C-8a), 128.28 (Ar-C), 129.03 (Ar-C), 129.65 (Ar-C), 130.09 (Ar-C), 130.92 (Ar-C), 138.15 (C-5), 140.24 (Ar-C), 151.89 (C-8b), 153.13 (C-14b), 154.00 (C-12), 160.09 (C-2), 162.03 (Ar-C), 165.50 (C-6a); MS (*m/z*), 474 (M^+^, 54.05) with a base peak at 279 (100); C_29_H_19_FN_4_O_2_ (474.49): calcd; % C: 73.41, % H: 4.04, % N: 11.81; found; % C: 73.48, % H: 4.11, % N: 11.98.

*14-(4-Chlorophenyl)-2-phenyl-12-methoxy-14H-benzo[h]chromeno[3,2-e][1,2,4]triazolo-[1,5-c]pyrimidine* (**7n**). Colourless needles. Yield: 66%; m.p. 295–296 °C; IR (KBr, cm*^−^*^1^): ν 3062, 3021 (CH-arom.), 2954, 2895 (CH-aliph.), 1621 (C=N); MS (*m/z*), 492 (M^+^ + 2, 3.13), 490 (M^+^, 9.42) with a base peak at 104 (100); C_29_H_19_ClN_4_O_2_ (490.94): calcd; % C: 70.95, % H: 3.90, % N: 11.41; found; % C: 71.00, % H: 3.94, % N: 11.45.

*14-(4-Bromophenyl)-2-phenyl-12-methoxy-14H-benzo[h]chromeno[3,2-e][1,2,4]triazolo-[1,5-c]pyrimidine* (**7o**). Colourless needles. Yield: 67%; m.p. 298–299 °C; IR (KBr, cm*^−^*^1^): ν 3046 (CH-arom.), 2979, 2945 (CH-aliph.), 1621 (C=N); MS (*m/z*), 536 (M^+^ + 2, 6.23), 534 (M^+^, 5.15) with a base peak at 433 (100); C_29_H_19_BrN_4_O_2_ (535.39): calcd; % C: 65.06, % H: 3.58, % N: 10.46; found; % C: 65.13, % H: 3.62, % N: 10.57.

#### 3.2.7. General Procedure for the Preparation of Compounds **8a**–**c**

A mixture of aminoimino compounds **6a**–**c** (3.88, 4.04, or 4.49 g, respectively, 0.01 mol) and benzaldehyde (1.06 g, 0.01mol) in ethanol (30 mL) and piperidine (0.5 mL) was refluxed for 2 h. The solvent was extracted and the resulting product was recrystallized from 1,4-dioxane to give the open chain products **8a**–**c**. The physical data of the compounds **8a**–**c** are as follows:

*9-Benzylideneamino-7-(4-fluorophenyl)-5-methoxy-8-imino-7H-benzo[h]chromeno[2,3-d]pyrimidine* (**8a**). Pale yellow crystals. Yield: 66%; m.p. 257–258 °C; IR (KBr, cm*^−^*^1^): ν 3204 (NH), 3055, 3000 (CH-arom.), 2951, 2850 (CH-aliph.), 1631 (C=N); ^1^H-NMR (DMSO-*d*_6_) δ 3.98 (s, 3H, OCH_3_), 6.13 (s, 1H, H-7), 6.80–8.26 (m, 14H, aromatic), 8.21 (s, 1H, H-10), 8.41 (N=CH), 11.03 (bs, 1H, NH); ^13^C-NMR (DMSO-*d*_6_) δ 38.52 (C-7), 56.19 (CH_3_), 96.78 (C-7a), 103.38 (C-6), 116.57 (Ar-C), 118.82 (C-6a), 121.74 (C-1), 124.01 (C-4), 124.54 (C-3), 126.33 (C-4a), 126.74 (C-2), 127.54 (C-1a), 128.91 (Ar-C), 129.62 (Ar-C), 130.23 (Ar-C), 132.64 (Ar-C), 134.64 (Ar-C), 137.55 (C-1b), 144.51 (C-5), 144.88 (Ar-C), 151.65 (C-11a), 156.54 (N=CH), 158.42 (C-10), 160.98 (Ar-C), 163.83 (C-8); MS (*m/z*), 476 (M^+^, 99.45) with a base peak at 380 (100); C_29_H_21_FN_4_O_2_ (476.50): calcd; % C: 73.10, % H: 4.44, % N: 11.76; found; % C: 73.14, % H: 4.47, % N: 11.81.

*9-Benzylideneamino-7-(4-chlorophenyl)-5-methoxy-8-imino-7H-benzo[h]chromeno[2,3-d]pyrimidine* (**8b**). Colourless powder. Yield: 86%; m.p. 260–261 °C; IR (KBr, cm*^−^*^1^): ν 3214 (NH), 3065, 3010 (CH-arom.), 2971, 2860 (CH-aliph.), 1629 (C=N); ^1^H-NMR (DMSO-*d*_6_) δ 3.97 (s, 3H, OCH_3_), 6.18 (s, 1H, H-7), 6.82–8.29 (m, 14H, aromatic), 8.21 (s, 1H, H-10), 8.42 (N=CH), 11.04 (bs, 1H, NH); ^13^C-NMR (DMSO-*d*_6_) δ 38.54 (C-7), 55.99 (CH_3_), 96.79 (C-7a), 103.36 (C-6), 118.80 (C-6a), 121.76 (C-1), 124.02 (C-4), 124.52 (C-3), 126.36 (C-4a), 126.50 (Ar-C), 126.76 (C-2), 127.56 (C-1a), 128.91 (Ar-C), 129.44 (Ar-C), 129.62 (Ar-C), 131.64 (Ar-C), 131.98 (Ar-C), 132.23 (Ar-C), 137.60 (C-1b), 144.53 (C-5), 146.88 (Ar-C),151.66 (C-11a), 156.58 (N=CH), 158.44 (C-10), 163.88 (C-8); MS (*m/z*), 494 (M^+^ + 2, 2.07), 492 (M^+^, 5.29) with a base peak at 385 (100); C_29_H_21_ClN_4_O_2_ (492.96): calcd; % C: 70.66, % H: 4.29, % N: 11.37; found; % C: 70.70, % H: 4.33, % N: 11.42.

*9-Benzylideneamino-7-(4-bromophenyl)-5-methoxy-8-imino-7H-benzo[h]chromeno[2,3-d]pyrimidine* (**8c**). Pale yellow crystals. Yield: 89%; m.p. 274–275 °C; IR (KBr, cm*^−^*^1^): ν 3216 (NH), 3040 (CH-arom.), 2937, 2894 (CH-aliph.), 1650 (C=N); ^1^H-NMR (DMSO-*d*_6_) δ 3.97 (s, 3H, OCH_3_)**,** 6.17 (s, 1H, H-7), 6.17–8.27 (m, 14H, aromatic), 8.28 (s, 1H, H-10), 8.44 (N=CH), 11.06 (bs, 1H, NH); ^13^C-NMR (DMSO-*d*_6_) δ 38.54 (C-7), 55.99 (CH_3_), 96.77 (C-7a), 103.37 (C-6), 118.80 (C-6a), 120.57(Ar-C), 121.73 (C-1), 124.03 (C-4), 124.53 (C-3), 126.35 (C-4a), 126.77 (C-2), 127.55(C-1a), 128.50 (Ar-C), 128.91 (Ar-C), 129.44 (Ar-C), 129.62 (Ar-C), 131.64 (Ar-C), 134.23 (Ar-C), 137.59 (C-1b), 143.88 (Ar-C), 144.54 (C-5), 151.68 (C-11a), 156.57 (N=CH), 158.45 (C-10), 163.87 (C-8); MS (*m/z*), 538 (M^+^ + 2, 4.61), 536 (M^+^, 4.70) with a base peak at 433 (100); C_29_H_21_BrN_4_O_2_ (537.41): calcd; % C: 64.81, % H: 3.94, % N: 10.43; found; % C: 64.77, % H: 3.99, % N: 10.48.

#### 3.2.8. Synthesis of Compounds **12a**–**c**

A mixture of aminoimino compounds **6a**–**c** (3.88, 4.04, or 4.49 g, respectively, 0.01 mol) and ethyl chloroformate (1.08 g, 0.01 mol) in dry benzene (30 mL) was refluxed for 2 h. The solid product was collected by filtration and recrystallized from ethanol/1,4-dioxane to give **12a**–**c**. The physical data of the compounds **12a**–**c** are as follows:

*3-Ethoxycarbonyl-14-(4-fluorophenyl)-12-methoxy-14H-benzo[h]chromeno[3,2-e][1,2,4]triazolo[1,5-c]-pyrimidine-2-one* (**12a**). Yellow crystals. Yield: 45%; m.p. 241–242 °C; IR (KBr, cm*^−^*^1^): ν 3051 (CH-arom.), 2983, 2950, 2880 (CH-aliph.), 1645 (C=N), 1721 (CO), 1770 (CO ester); ^1^H-NMR (DMSO-*d*_6_) δ 1.35 (t, 3H, CH_3_, *J =* 7.2 Hz), 3.89 (s, 3H, OCH_3_), 4.44 (q, 2H, CH_2_, *J =* 7.2 Hz), 5.59 (s, 1H, H-14), 6.82–8.29 (m, 9H, aromatic), 9.35 (s, 1H, H-5); ^13^C-NMR (DMSO-*d*_6_) δ 13.96 (CH_3_), 40.03 (C-14), 55.92 (CH_3_), 64.47 (CH_2_), 98.45 (C-14a), 103.55 (C-13), 115.38 (Ar-C), 117.65 (C-13a), 120.51 (C-8), 121.79 (C-11), 123.88 (C-10), 124.63 (C-11a), 127.84 (C-9), 130.14 (C-8a), 130.19 (Ar-C), 137.46 (C-8b), 139.59 (Ar-C), 140.51 (C-5), 148.77 (C-12), 152.21 (C-6a), 155.11 (Ar-C), 155.21 (C-14b), 157.39 (CO), 160.32 (CO); MS (*m/z*), 486 (M^+^, 2.44) with a base peak at 320 (100); C_26_H_19_FN_4_O_5_ (486.45): calcd; % C: 64.20, % H: 3.94, % N: 11.52; found; % C: 64.24, % H: 3.99, % N: 11.56.

*3-Ethoxycarbonyl-14-(4-chlorophenyl)-12-methoxy-14H-benzo[h]chromeno[3,2-e][1,2,4]triazolo[1,5-c]-pyrimidine-2-one* (**12b**). Colourless needles. Yield: 43%; m.p. 200–201 °C; IR (KBr, cm*^−^*^1^): ν 3020 (CH-arom.), 2992, 2970, 2860 (CH-aliph.), 1641 (C=N), 1740 (CO), 1759 (CO ester); ^1^H-NMR (DMSO-*d*_6_) δ 1.35 (t, 3H, CH_3_, *J =* 7.2 Hz), 3.89 (s, 3H, OCH_3_), 4.44 (q, 2H, CH_2_, *J =* 7.2 Hz), 5.59 (s, 1H, H-14), 6.82–8.29 (m, 9H, aromatic), 9.35 (s, 1H, H-5); ^13^C-NMR (DMSO-*d*_6_) δ 13.98 (CH_3_), 40.04 (C-14), 55.94 (CH_3_), 64.50 (CH_2_), 98.38 (C-14a), 103.51 (C-13), 117.33 (C-13a), 120.53 (C-8), 121.81 (C-11), 123.89 (C-10), 124.69 (C-11a), 126.70 (Ar-C), 127.88 (C-9), 128.55 (C-8a), 130.15 (Ar-C), 131.78 (Ar-C), 137.49 (C-8b), 140.59 (C-5), 142.29 (Ar-C), 148.80 (C-12), 152.25 (C-6a), 155.12 (C-14b), 155.23 (CO), 157.40 (CO); MS (*m/z*), 504 (M^+^ + 2, 5.24), 502 (M^+^, 16.36) with a base peak at 320 (100); C_26_H_19_ClN_4_O_5_ (502.91): calcd; % C: 62.09, % H: 3.81, % N: 11.14; found; % C: 62.03, % H: 3.77, % N: 11.17.

*3-Ethoxycarbonyl-14-(4-bromophenyl)-12-methoxy-14H-benzo[h]chromeno[3,2-e][1,2,4]-triazolo[1,5-c]pyrimidine-2-one* (**12c**). Colourless powder. Yield: 49%; m.p. 200–201 °C; IR (KBr, cm*^−^*^1^): ν 3068 (CH-arom.), 2975, 2937, 2885 (CH-aliph.), 1646 (C=N), 1736 (CO), 1773 (CO ester); ^1^H-NMR (DMSO-*d*_6_) δ 1.34 (t, 3H, CH_3_, *J =* 7.2 Hz), 3.89 (s, 3H, OCH_3_), 4.44 (q, 2H, CH_2_, *J =* 7.2 Hz), 5.57 (s, 1H, H-14), 6.81–8.28 (m,9H, aromatic), 9.35 (s, 1H, H-5); ^13^C-NMR (DMSO-*d*_6_) δ 13.98 (CH_3_), 40.05 (C-14), 55.93 (CH_3_), 64.50 (CH_2_), 98.31 (C-14a), 103.49 (C-13), 117.23 (C-13a), 120.53 (Ar-C), 121.81 (C-8), 123.87 (C-11), 124.68 (C-10), 126.69 (C-11a), 127.78 (C-9), 130.32 (C-8a), 130.52 (Ar-C), 131.46 (Ar-C), 137.48 (C-8b), 140.58 (C-5), 142.70 (Ar-C), 148.79 (C-12), 152.25 (C-6a), 155.11 (C-14b), 155.21 (CO), 157.37 (CO); MS (*m/z*), 548 (M^+^ + 2, 10.14), 546 (M^+^, 10.90) with a base peak at 320 (100); C_26_H_19_BrN_4_O_5_ (547.36): calcd; % C: 57.05, % H: 3.50, % N: 10.24; found; % C: 57.11, % H: 3.55, % N: 10.27.

### 3.3. Antimicrobial Assay

All the newly synthesized compounds **7a**–**o**, **8a**–**c** and **12a**–**c** were screened for their in vitro antimicrobial activity at 25 µg/mL to determine the zone of inhibition against four Gram-positive bacteria: *Staphylococcus aureus* (RCMB 000106), *Staphylococcus epidermidis* (RCMB 000107), *Bacillis subtilis* (RCMB 000108), *Bacillus pumilus* (RCMB 000109), and two Gram-negative pathogenic bacteria: *Pseudomonas aeruginosa* (RCMB 000102), *Escherichia coli* (RCMB 000103) using two standard antibiotics (ampicillin, streptomycin) as reference drugs, and three fungi: *Aspergillus fumigatus* (RCMB 002003), *Candida albicans* (RCMB 005002) and *Saccharomyces cerevisiae* (RCMB 006002) using two standard antibiotics (mycostatine, clotrimazole) as reference drugs. The activities of these compounds were tested by agar diffusion method using Mueller-Hinton agar medium for bacteria and Sabouraud’s agar medium for fungi [31,32]. The tested compounds were dissolved in *N,N*-dimethylformamide (DMF) to give a solution of 1 mg·mL^−1^. The inhibition zones (diameter of the hole) were measured in millimeters (6 mm) at the end of an incubation period of 48 h at 28 °C; *N,N*-dimethylformamide showed no inhibition zone.

## 4. Conclusions

In conclusion, several 14-(4-halophenyl)-12-methoxy-14*H*-benzo[*h*]chromeno[3,2-*e*][1,2,4]-triazolo[1,5-*c*]pyrimidine and 7*H*-benzo[*h*]chromeno[2,3-*d*]pyrimidine derivatives were synthesized in good yields, starting from 4*H*-benzo[*h*]chromene derivatives. The structures of compounds **7**, **8** and **12** were established on the basis of IR, ^1^H-NMR, ^13^C-NMR and MS data. A pharmacological study has been performed in order to evaluate the effects of substituents on the antibacterial and antifungal activities. Compounds **12a**–**c** did not show any antimicrobial activity against any of the tested bacteria and fungi, while compounds **7a**,**i**,**h**,**g**,**m**,**b**,**n**,**d**,**o**,**c**,**e**,**f** showed high to good activities only against *Staphylococcus epidermidis* as compared to ampicillin and streptomycin. In contrast, compounds **7j**–**l** and **8a**–**b** showed high activity against all tested microorganisms. The rest of compounds showed almost equipotent and moderate activities or were inactive. The structure**-**activity relationship (SAR) study revealed that the antimicrobial activity of 14*H*-benzo[*h*]chromeno[3,2-*e*][1,2,4]triazolo[1,5-*c*]-pyrimidine nucleus was more affected by the lipophilicity of the non-substituent or 2-substituents than by lipophilic hydrophobic groups (=NH-8, –N=CHPh-9) on the 7*H*-benzo[*h*]-chromeno[2,3-*d*]pyrimidine nucleus and the 14*H*-benzo[*h*]chromeno[3,2-*e*][1,2,4]triazolo[1,5-*c*]-pyrimidine nucleus was more beneficial than the 7*H*-benzo[*h*]chromeno[2,3-*d*]pyrimidine nucleus for the antimicrobial activity.

## Figures and Tables

**Table 1:**
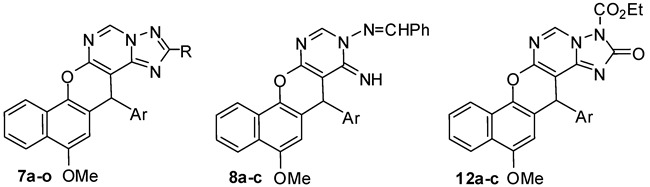
Antimicrobial screening for compounds **7a**–**o**, **8a**–**c** and **12a**–**c** (25 µg/mL).

Compound	R	Ar	*S. aureus* (+ve)	*S. Epider.* (+ve)	*B. Subtilis* (+ve)	*B. pumilus* (+ve)	P. *Aerug.* (−ve)	*E. coli* (−ve)	*A. fumig.*	*C. albic.*	*S. cerev.*
**7a**	H	4-FC_6_H_5_	NA	11.9 ± 0.3	NA	NA	NA	NA	NA	NA	NA
**7b**	H	4-ClC_6_H_5_	NA	20.5 ± 0.2	NA	NA	NA	NA	NA	NA	NA
**7c**	H	4-BrC_6_H_5_	NA	23.5 ± 0.1	NA	NA	NA	NA	NA	NA	NA
**7d**	CH_3_	4-FC_6_H_5_	NA	21.1 ± 0.1	NA	NA	NA	NA	NA	NA	NA
**7e**	CH_3_	4-ClC_6_H_5_	NA	23.5 ± 0.1	NA	NA	NA	NA	NA	NA	NA
**7f**	CH_3_	4-BrC_6_H_5_	NA	24.3 ± 0.1	NA	NA	NA	NA	NA	NA	NA
**7g**	CO_2_Et	4-FC_6_H_5_	NA	18.9 ± 0.1	NA	NA	NA	NA	NA	NA	NA
**7h**	CO_2_Et	4-ClC_6_H_5_	NA	17.2 ± 0.2	NA	NA	NA	NA	NA	NA	NA
**7i**	CO_2_Et	4-BrC_6_H_5_	NA	16.1 ± 0.3	NA	NA	NA	NA	NA	NA	NA
**7j**	CH_2_CN	4-FC_6_H_5_	27.3 ± 0.5	25.2 ± 0.1	12.3 ± 0.3	12.3 ± 0.3	28.3 ± 0.07	26.7 ± 0.1	12.3 ± 0.3	19.3 ± 0.1	23.3 ± 0.1
**7k**	CH_2_CN	4-ClC_6_H_5_	28.8 ± 0.1	26.3 ± 0.3	12.2 ± 0.4	12.2 ± 0.4	28.2 ± 0.03	26.8 ± 0.3	12.2 ± 0.1	21.2 ± 0.2	20.2 ± 0.1
**7l**	CH_2_CN	4-BrC_6_H_5_	29.1 ± 0.2	26.9 ± 0.3	12.7 ± 0.4	12.8 ± 0.4	28.9 ± 0.03	27.8 ± 0.3	12.1 ± 0.2	21.1 ± 0.2	18.2 ± 0.1
**7m**	Ph	4-FC_6_H_5_	NA	19.3 ± 0.5	NA	NA	NA	NA	NA	NA	NA
**7n**	Ph	4-ClC_6_H_5_	NA	20.9 ± 0.1	NA	NA	NA	NA	NA	NA	NA
**7o**	Ph	4-BrC_6_H_5_	NA	21.4 ± 0.1	NA	NA	NA	NA	NA	NA	NA
**8a**	-	4-FC_6_H_5_	21.8 ± 0.1	22.9 ± 0.1	23.1 ± 0.2	24.0 ± 0.3	19.3 ± 0.1	21.9 ± 0.1	23.8 ± 0.1	22.9 ± 0.1	16.8 ± 0.4
**8b**	-	4-ClC_6_H_5_	24.6 ± 0.2	25.7 ± 0.2	25.1 ± 0.4	23.6 ± 0.1	21.3 ± 0.2	21.0 ± 0.4	23.4 ± 0.1	22.4 ± 0.1	18.2 ± 0.3
**8c**	-	4-BrC_6_H_5_	26.6 ± 0.1	28.5 ± 0.3	25.1 ± 0.4	21.3 ± 0.1	23.3 ± 0.1	20.7 ± 0.2	22.1 ± 0.1	21.7 ± 0.2	19.5 ± 0.1
**12a**	-	4-FC_6_H_5_	NA	NA	NA	NA	NA	NA	NA	NA	NA
**12b**	-	4-ClC_6_H_5_	NA	NA	NA	NA	NA	NA	NA	NA	NA
**12c**	-	4-BrC_6_H_5_	NA	NA	NA	NA	NA	NA	NA	NA	NA
Ampicillin	-	-	27.4 ± 0.1	28.6 ± 0.5	29.4 ± 0.7	29.9 ± 0.3	26.3 ± 0.3	28.5 ± 0.1	-	-	-
Streptomycin	-	-	25.1 ± 0.2	29.1 ± 0.3	30.1 ± 0.9	30.8 ± 0.4	24.3 ± 0.8	25.6 ± 0.4	-	-	-
Mycostatine	-	-	-	-	-	-	-	-	29.5 ± 0.1	27.1 ± 0.1	24.1 ± 0.3
Clotrimazole	-	-	-	-	-	-	-	-	26.1 ± 0.1	23.1 ± 0.3	18.3 ± 0.1

NA = not active; Diameter of the hole = 6 mm; Data are expressed in the form of mean ± SD.

## References

[1] Saini M.S., Kumar A., Dwivedi J., Singh R. (2013). A Review: Biological Significances of Heterocyclic Compounds. Int. J. Pharm. Sci. Res..

[2] Li Q.-Z., Nie X.-Y., Liang J. (2011). Novel Coumarin and 4*H*-Chromene Derivatives Containing 4,5-Dihydropyrazole Moiety: Synthesis and Antibacterial Activity. Lett. Drug Des. Discov..

[3] Liu X.-H., Liu J.-X., Bai L.-S., Lan G.-L., Pan C.-X. (2010). Novel dihydropyrazole Derivatives Linked with 4*H*-Chromene: Microwave-Promoted Synthesis and Antibacterial Activity. Lett. Drug. Des. Discov..

[4] Foroumadi A., Emami S., Sorkhi M., Nakhjiri M., Nazarian Z., Heydar S., Ardestani S., Poorrajab F., Shafiee A. (2010). Chromene-Based Synthetic Chalcones as Potent Antileishmanial Agents: Synthesis and Biological Activity. Chem. Biol. Drug Des..

[5] Narender T., Shweta Gupta S. (2009). A convenient and biogenetic type synthesis of few naturally occurring chromeno dihydrochalcones and their in vitro antileishmanial activity. Bioorg. Med. Chem. Lett..

[6] Sabry N.M., Mohamed H.M., Khattab E.S.A.E.H., Motlaq S.S., El-Agrody A.M. (2011). Synthesis of 4*H* chromene, coumarin, 12*H*-chromeno[2,3-*d*]pyrimidine derivatives and some of their antimicrobial and cytotoxicity activities. Eur. J. Med. Chem..

[7] Rampa A., Bisi A., Belluti F., Gobbi S., Piazzi L., Valenti P., Zampiron A., Caputo A., Varani K., Borea P.A., Carrara M. (2005). Homopterocarpanes as bridged triarylethylene analogues:Synthesis and antagonistic effects in human MCF-7 breast cancer cells. IL Farmco.

[8] Magedov I.V., Manpadi M., Evdokimov N.M., Elias E.M., Rozhkova E., Ogasawara M.A., Bettale J.D., Przheval’skii N.M., Rogelj S, Kornienko A. (2007). Antiproliferative and apoptosis inducing properties of pyrano[3,2-*c*]pyridones accessible by a onestep multicomponent synthesis. Bioorg. Med Chem. Lett..

[9] Singh O.M., Devi N.S., Thokchom D.S., Sharma G.J. (2010). Novel 3-alkanoyl/aroyl/heteroaroyl-2*H*-chromene-2-thiones: Synthesis and evaluation of their antioxidant activities. Eur. J. Med. Chem..

[10] Vukovic N., Sukdolak S., Solujic S., Niciforovic N. (2010). Substituted imino and amino derivatives of 4-hydroxycoumarins as novel antioxidant, antibacterial and antifungal agents: synthesis and in vitro assessments. Food Chem..

[11] Tandon V.K., Vaish M., Jain S., Bhakuni D.S., Srimal R.C. (1991). Synthesis, carbon-13 NMR and hypotensive action of 2,3-dihydro-2,2-dimethyl-4*H*-naphtho [1,2-*b*] pyran-4-one. Indian J. Pharm. Sci..

[12] Mahmoodi M., Aliabadi A., Emami S., Safavi M., Rajabalian S., Mohagheghi M.A., Khoshzaban A., Samzadeh-Kermani A., Lamei N., Shafiee A. (2010). Synthesis and in vitro Cytotoxicity of Polyfunctionalized 4-(2-Arylthiazol-4-yl)-4*H*-Chromemes. Arch. Pharm. Chem..

[13] Endo S., Matsunaga T., Kuwata K., Zhao H.-T., El-Kabbani O., Kitade Y., Hara A. (2010). Chromene-3-carboxamide derivatives discov ered from virtual screening as potent inhibitors of the tumor maker, AKR1B10. Bioorg. Med. Chem..

[14] Tseng T.-H., Chuang S.-K., Hu C.-C., Chang C.-F., Huang Y.-C., Lin C.-W., Lee Y.-J. (2010). The synthesis of morusin as a potent antitumor agent. Tetrahedron.

[15] El-Agrody A.M., Fouda A.M., Khattab E.S.A.E.H. (2013). Synthesis, antitumor activity of 2-amino-4*H*-benzo[*h*]chromene derivatives and Structure-activity relationships of the 3- and 4-positions. Med. Chem. Res..

[16] Bruhlmann C., Ooms F., Carrupt P., Testa B., Catto M., Leonetti F., Altomare C., Cartti A. (2001). Coumarins derivatives as dual inhibitors of acetylcholinesterase and monoamine oxidase. J. Med. Chem..

[17] Kesten S.R., Heffner T.G., Johnson S.J., Pugsley T.A., Wright J.L., Wise D.L. (1999). Design, Synthesis, and Evaluation of Chromen-2- ones as Potent and Selective Human Dopamine D4 Antagonists. J. Med. Chem..

[18] Lee K.-S., Khil L.-Y., Chae S.-H., Kim D., Lee B.-H., Hwang G.-S., Moon C.-H., Chang T.-S., Moon C.-K. (2006). Effects of DK-002, a synthesized(6aS,*cis*)-9,10-Dimethoxy-7,11b-dihydro-indeno[2,1-*c*]chromene-3,6a-diol, on platelet activity. Life Sci..

[19] Coudert P., Coyquelet J.M., Bastide J., Marion Y., Fialip J. (1988). Synthesis and anti-allergic properties of *N*-arylnitrones with furo-pyran structure. Ann. Pharm. Fr..

[20] El-Sayed A.T., Ibrahim M.A. (2010). Synthesis and Antimicrobial Activity of Chromone- linked-2-Pyridone Fused with 1,2,4-Triazoles, 1,2,4-Triazines and 1,2,4-Triazepines Ring Systems. J. Braz. Chem..

[21] Narasimhan B., Kumar P., Sharma D. (2010). Biological activities of hydrazide derivatives in the new millennium. Acta Pharm. Sci..

[22] Keri R.S., Hosamani K.M., Shingalapur R.V., Hugar M.H. (2010). Analgesic, anti-pyretic and DNA cleavage studies of novel pyrimidine derivatives of coumarin moiety. Eur. J. Med. Chem..

[23] Sashidhara K.V., Kumar M., Modukuri R.K., Srivastava A., Puri A. (2011). Discovery and synthesis of novel substituted benzocoumarins as orally active lipid modulating agents. Bioorg. Med Chem. Lett..

[24] Hiramoto K., Nasuhara A., Michiloshi K., Kato T., Kikugawa K. (1997). DNA strand-breaking activity and mutagenicity of 2,3-dihydro-3,5-dihydroxy-6-methyl-4*H*-pyran-4-one (DDMP), a Maillard reaction product of glucose and glycine. Mutat. Res..

[25] El-Agrody A.M., El-Hakium M.H., Abd El-Latif M.S., Fekry A.H., Sayed S.M., El-Gareab K.A. (2000). Synthesis of Pyrano[2,3-*d*]pyrimidine and Pyrano[3,2-*e*][1,2,4]triazolo[2,3-*c*]pyri- midine Derivatives with Promising Antimicrobial Activities. Acta Pharm..

[26] Bedair A.H., Emam H.A., El-Hady N.A., Ahmed K.A.R., El-Agrody A.M. (2001). Synthesis and Antimicrobial Activities of Novel Naphtho[2,1-*b*]pyran, Pyrano[3,2-*d*]pyrimidine and Pyrano-[3,2-*e*][1,2,4]triazolo-[2,3-*c*]pyrimidine Derivatives. Il Farmaco.

[27] Khafagy M.M., Abd El-Wahab A.H.F., Eid F.A., El-Agrody A.M. (2002). Synthesis of Halogen Derivatives of Benzo[*h*]cheromene and Benzo[*a*]anthracene with Promising Antimicrobial Activities. Il Farmaco.

[28] El-Agrody A.M., Sabry N.M., Motlaq S.S. (2011). Synthesis of some new 2-substituted 12*H*-chromeno[3,2-*e*][1,2,4]triazolo[1,5-*c*]pyrimidine, 3-ethoxycarbonyl-12*H*-chromeno[3,2-*e*]-[3,2-*e*][1,2,4]triazolo[1,5-*c*]pyrimidine-2-one, ethyl 2-formylamino/acetylamino-4*H*-chromene-3-carboxylate and some of their ntimicrobial activities. J. Chem. Res..

[29] Halawa A.H., Fouda A.M., Al-Dies A.M., El-Agrody A.M. (2016). Synthesis, Biological Evaluation and Molecular Docking Studies of 4*H*benzo[*h*]chromenes, 7*H*-benzo[*h*]chromeno-[2,3-*d*]pyrimidines as Antitumor Agents. Lett. Drug. Des. Discov..

[30] El-Agrody A.M., Halawa A.H., Fouda A.M., Al-Dies A.M. (2016). The anti-proliferative activity of novel 4*H*-benzo[*h*]chromenes, 7*H*-benzo[*h*]chromeno[2,3-*d*]pyrimidines and the structure-activity relationships of the 2-,3-positions and fused rings at the 2,3-positions. J. Saudi Chem. Soc..

[31] El-Agrody A.M., Fouda A.M., Al-Dies A.M. (2014). Studies on the synthesis, in vitro antitumor activity of 4*H*-benzochromene, 7*H*-benzo[*h*]chromeno[2,3-*d*]pyrimidine derivatives and Structure activity relationships of the 2-,3- and 2,3-positions. Med. Chem. Res..

[32] European Committee for Antimicrobial Susceptibility Testing (EUCAST) of the European Society of Clinical Microbiology and Infectious Diseases (ESCMID) (2000). Determination of minimum inhibitory concentrations (MICs) of antibacterial agents by agar dilution. Clin. Microbiol. Infect..

[33] National Committee for Clinical Laboratory Standards (2000). Methods for Dilution Antimicrobial Susceptibility Tests for Bacteria That Grow Aerobically.

[34] Al-Dies A.M., Amr A.-G.E., El-Agrody A.M., Chia T.S., Fun H.-K. (2012). 2-Amino-4-(4-fluorophenyl)-6-methoxy-4*H*-benzo[*h*]chromene-3-carbonitrile. Acta Cryst..

[35] Al-Sehemi A.G., Irfan A., El-Agrody A.M. (2012). Synthesis, characterization and DFT study of 4*H*-benzo[*h*]chromene derivatives. J. Mol. Struct..

[36] Al-Dies A.M., El-Agrody A.M., Al-Omar M.A., Amr A.-G.E., Ng S.W., Tiekink E.R.T. (2013). 2-Amino-4-(4-bromophenyl)-6-methoxy-4*H*-benzo[*h*]chromene-3-carbonitrile. Acta Cryst..

[37] El-Agrody A.M., Al-Dies A.M., Fouda A.M. (2014). Microwave assisted synthesis of 2-amino-6-methoxy-4*H*-benzo[*h*]chromene derivatives. Eur. J. Chem..

